# Argatroban versus heparin in patients without heparin-induced thrombocytopenia during venovenous extracorporeal membrane oxygenation: a propensity-score matched study

**DOI:** 10.1186/s13054-021-03581-x

**Published:** 2021-04-29

**Authors:** Christoph Fisser, Maren Winkler, Maximilian V. Malfertheiner, Alois Philipp, Maik Foltan, Dirk Lunz, Florian Zeman, Lars S. Maier, Matthias Lubnow, Thomas Müller

**Affiliations:** 1 Department of Internal Medicine II, University Medical Center Regensburg, Franz-Josef-Strauß-Allee 11, 93053 Regensburg, Germany; 2 Department of Cardiothoracic Surgery, University Medical Center Regensburg, Regensburg, Germany; 3 Department of Anesthesiology, University Medical Center Regensburg, Regensburg, Germany; 4 Center for Clinical Studies, University Medical Center Regensburg, Regensburg, Germany

**Keywords:** ECMO, Anticoagulation, Argatroban, Heparin, Thrombosis, Bleeding, Costs

## Abstract

**Background:**

During venovenous extracorporeal membrane oxygenation (vvECMO), direct thrombin inhibitors are considered by some potentially advantageous over unfractionated heparin (UFH). We tested the hypothesis that Argatroban is non-inferior to UFH regarding thrombosis and bleeding during vvECMO.

**Methods:**

We conducted a propensity-score matched observational non-inferiority study of consecutive patients without heparin-induced-thrombocytopenia (HIT) on vvECMO, treated between January 2006 and March 2019 in the medical intensive care unit at the University Hospital Regensburg. Anticoagulation was realized with UFH until August 2017 and with Argatroban from September 2017 onwards. Target activated partial thromboplastin time was 50 ± 5seconds in both groups. Primary composite endpoint was major thrombosis and/or major bleeding. Major bleeding was defined as a drop in hemoglobin of ≥ 2 g/dl/day or in transfusion of ≥ 2 packed red cells/24 h, or retroperitoneal, cerebral, or pulmonary bleeding. Major thrombosis was defined as obstruction of > 50% of the vessel lumen diameter by means of duplex sonography. We also assessed technical complications such as oxygenator defects or pump head thrombosis, the time-course of platelets, and the cost of anticoagulation (including HIT-testing).

**Results:**

Out of 465 patients receiving UFH, 78 were matched to 39 patients receiving Argatroban. The primary endpoint occurred in 79% of patients in the Argatroban group and in 83% in the UFH group (non-inferiority for Argatroban, *p* = 0.026). The occurrence of technical complications was equally distributed (Argatroban 49% vs. UFH 42%, *p* = 0.511). The number of platelets was similar in both groups before ECMO therapy but lower in the UFH group after end of ECMO support (median [IQR]: 141 [104;198]/nl vs. 107 [54;171]/nl, *p* = 0.010). Anticoagulation costs per day of ECMO were higher in the Argatroban group (€26 [13.8;53.0] vs. €0.9 [0.5;1.5], *p* < 0.001) but not after accounting for blood products and HIT-testing (€63 [42;171) vs. €40 [17;158], *p* = 0.074).

**Conclusion:**

In patients without HIT on vvECMO, Argatroban was non-inferior to UFH regarding bleeding and thrombosis. The occurrence of technical complications was similarly distributed. Argatroban may have less impact on platelet decrease during ECMO, but this finding needs further evaluation. Direct drug costs were higher for Argatroban but comparable to UFH after accounting for HIT-testing and transfusions.

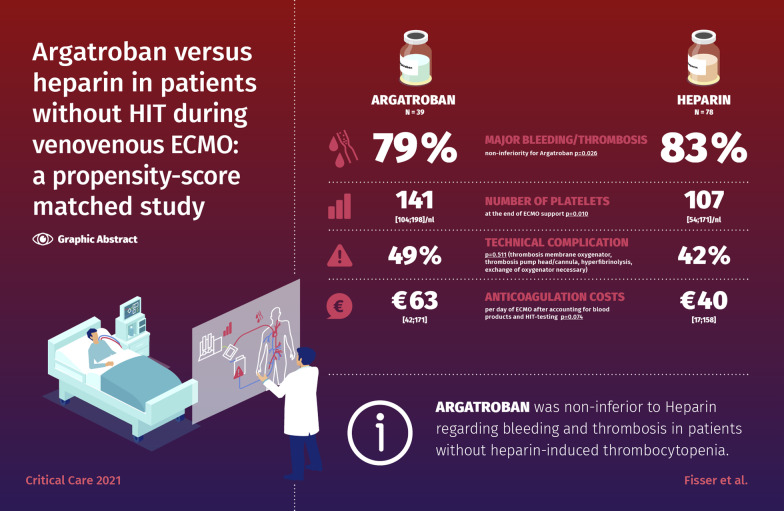

**Supplementary Information:**

The online version contains supplementary material available at 10.1186/s13054-021-03581-x.

## Introduction

Since the first successful application of extracorporeal membrane oxygenation (ECMO) in 1971 [1], the use of ECMO has been steadily increasing, particularly that of veno-venous ECMO (vvECMO) during the 2009–2010 H1N1 influenza-A pandemic [2] and the recent COVID-19 pandemic [3]. Despite important improvements and technical developments, bleeding and thrombosis as side effects of ECMO therapy [4] are still very common and have a critical impact on outcome. Due to the activation of the coagulation cascade by nonendothelial artificial surfaces [5], unfractionated heparin (UFH) is the anticoagulation regime currently recommended by the Extracorporeal Life Support Organization (ELSO) [5]. However, alternative anticoagulation strategies are being investigated [6], and direct thrombin inhibitors (DTI) have been proposed to have potential advantages. In particular, some scientists consider DTIs such as Argatroban to be superior to UFH because of their capacity to directly bind to the active site of thrombin, either circulating or clot bound [7]. In contrast to heparin, antithrombin is not needed for the anticoagulatory effect of DTI, probably making its efficacy more reliable across mixed populations [6]. Fast metabolization with a short half-life time of around 15 min is of additionally value for patients on ECMO support who have an increased risk of bleeding [6]. When using Argatroban as anticoagulant in critically ill patients, specific attention has to be paid to the initial requirement of a much lower dosage to impede bleeding events. Furthermore, the hepatic metabolization, which forbids to use Argatroban in liver failure, has to be taken into account [8].

A distinct disadvantage of UFH is the risk of type II heparin-induced thrombocytopenia (HIT), which has been reported in up to 4% of patients on ECMO support [9]. HIT results in high rates of thrombosis and increases mortality [10]. The application of DTIs such as Argatroban has improved survival in patients with HIT who do not require ECMO support [11]. In ECMO management, Argatroban has so far only been used in a small number of patients with confirmed HIT [8, 12]. Knowledge about the pros and cons of the different anticoagulation substances in clinical ECMO practice is still scarce, and comparative studies are lacking.

In this context, the aim of this study was to investigate if Argatroban is non-inferior to UFH with respect to the occurrence of thrombosis and bleeding in patients without HIT during vvECMO support. Additional analyses included the efficacy and costs of anticoagulation as well as technical complications.

## Material and methods

### Study subjects

**Fig. 1:**
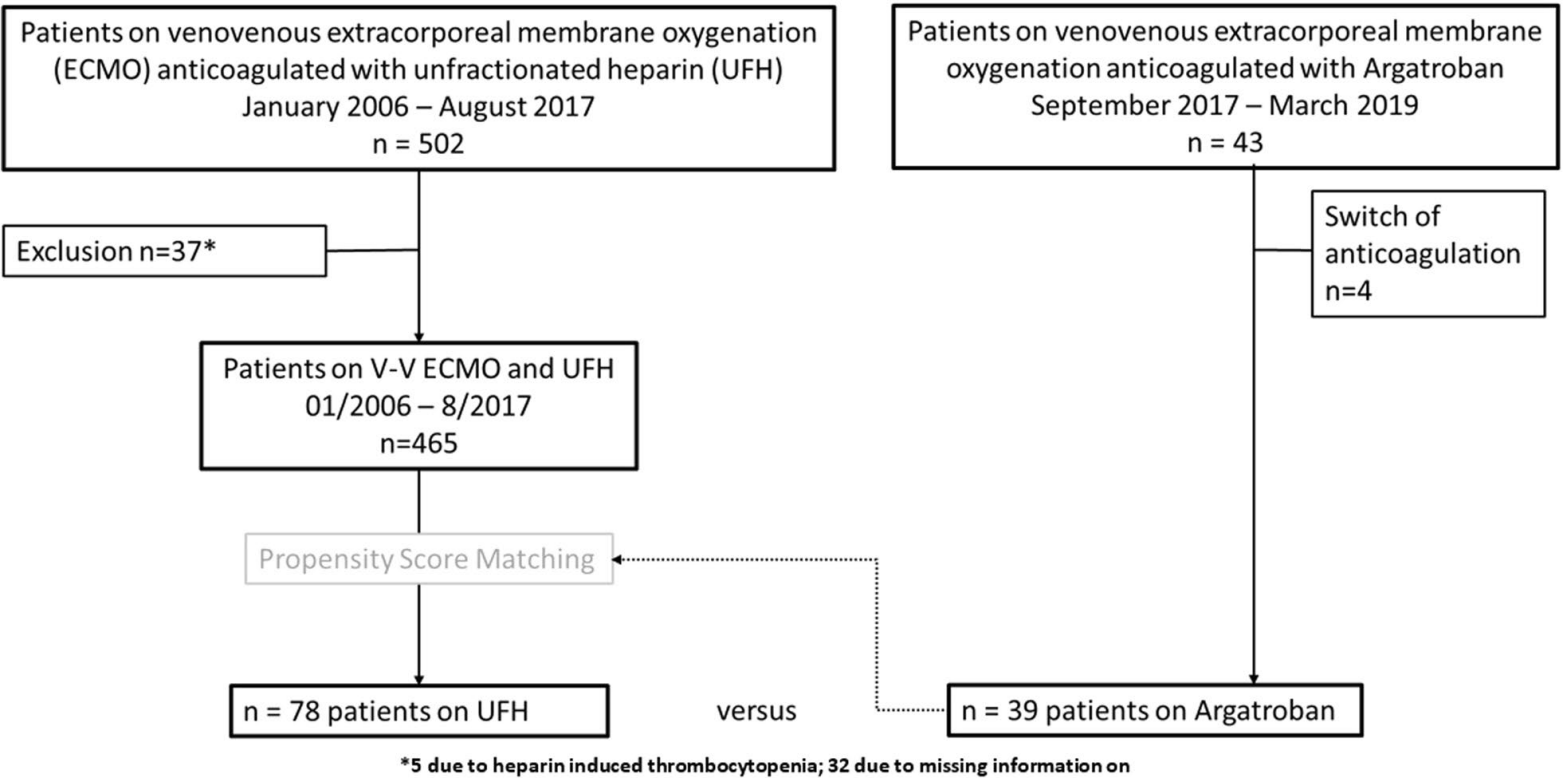
Flowchart of the observational study of the prospective extracorporeal membrane oxygenation (ECMO) registry Regensburg

This analysis included all patients with severe respiratory failure (PaO_2_/FiO_2_ < 85 mmHg or refractory respiratory acidosis with pH < 7.25 on optimized PEEP, usually ≥ 15 cmH_2_O) of the Regensburg ECMO-Registry who had received vvECMO in an intensive care unit of the University Medical Center Regensburg between August 2006 and March 2019. In September 2017, the standard anticoagulation regime was switched from UFH to Argatroban (Fig. 1). All patients who had received UFH and had shown an unexplained decline in platelets of ≥ 30% after more than four days of ECMO therapy were routinely screened for HIT. Patients with HIT confirmed by ELISA (antibodies against platelet factor 4; HemosIL Acustar HIT-IgG, Werfen, Germany) and HIPA (heparin-induced platelet aggregation; Department of Immunological and Transfusion Medicine, University Hospital Greifswald) were excluded from the analysis.

Routine data such as demographics, daily laboratory parameters, and disease scores were extracted from the electronic patient data management system of our hospital. The total doses of anticoagulation were recorded. Disseminated intravascular coagulation (DIC) was defined according to the International Society on Thrombosis and Hemostasis DIC score [13]. Further details on the DIC score are provided in the supplement (Additional file 1: Table e1) and have been published previously [4].

The study protocol was reviewed and approved by the local institutional Ethics Committee (19-1335-104). The study was conducted according to the Declaration of Helsinki on Good Clinical Practice.

### Anticoagulation strategy during ECMO support

Before September 2017, UFH was used according to our institutional standard anticoagulation protocol for patients on vvECMO support. After September 2017, Argatroban was used as a first line anticoagulation strategy with an initial infusion rate of 0.2 µg/kg/min [8]. Target activated partial thromboplastin time (aPTT) was 50 ± 5 s in both groups [8, 12]. Argatroban concentration was measured once daily in the Argatroban group. Further details on the anticoagulation strategy are provided in the supplemental material (Additional file 1: Tables e2–4).

### Study outcomes

The primary end point was the occurrence of major bleeding and/or major thrombosis. Major bleeding was defined according to the recommendations by the ELSO [5] as a drop in hemoglobin of ≥ 2 g/dl/day or in transfusion of ≥ 2 packed red cells/24 h, or retroperitoneal, cerebral, or pulmonary bleeding. Minor bleeding was defined as < 2 packed red cells/24 h. In addition, physicians’ documentation (once per day) and nurses’ shift reports (3 times per day) were evaluated for bleeding events. 

Deep vein thrombosis in the area of cannulation (incompressibility of the vein and absence or reduction of flow) was diagnosed by specially trained physicians by means of duplex sonography after decannulation [14, 15]. Obstruction of > 50% of the vessel lumen diameter was classified as major thrombosis and obstruction of ≤ 50% as minor thrombosis [4]. All CT scans, which were performed for clinical reasons during or after ECMO, were assessed for pulmonary embolism.

Secondary end points included major thrombosis, major bleeding, the time-course of the platelet counts, the cumulative transfusion of blood products (red blood cells [RBC], fresh frozen plasma [FFP], and platelets), and the incidence of intracranial bleeding. Cerebral hemorrhage was assessed by means of a CT scan. The absolute number of platelets was documented daily for the entire duration of ECMO support. Additional secondary endpoints were technical and clinical complications such as thrombosis of the membrane oxygenator or pump head, occurrence of hyperfibrinolysis requiring oxygenator exchange according to the criteria previously defined by the ELSO [16], as well as the direct costs of drugs, blood products, and HIT diagnostics. Further information on cost analysis is provided in the supplements (Additional file 1: Table e5).

### Statistics

Propensity-score matching was carried out by matching every patient who received Argatroban with a historical UFH patient to control for potential confounders such as age, sex, body mass index (BMI), sepsis-related organ failure assessment (SOFA), the acute respiratory distress syndrome (ARDS) subgroup (pulmonary, non-pulmonary, post-trauma, and miscellaneous), resuscitation and days of mechanical ventilation before ECMO, and days on ECMO to account for potential imbalances between the Argatroban and the UFH group. More details are presented in the supplements.

All non-normally distributed quantitative data are expressed as median (interquartile range) and were compared with the Mann–Whitney-U test. Differences between the two study groups were assessed with the Chi-squared test of independence for nominal variables. Linear regression models were calculated to assess the association between the Argatroban plasma concentration and aPTT or SOFA. All reported *p* values were two-sided, and a *p* value of ≤ 0.05 was considered statistically significant. Data entry and calculation were done using Microsoft EXCEL365 ProPlus (Microsoft, USA), IBM SPSS Statistic software version 25.0 (SPSS Inc. Chicago, IL, USA), and the software package R (v 3.6.1 R Foundation for Statistical Computing).

## Results

### Study population

From September 2017 to March 2019, 43 patients on vvECMO support had received Argatroban for anticoagulation. Four patients had to be excluded from analysis due to receiving a second anticoagulant during ECMO support; thus, our Argatroban group included 39 patients. Our control group included 465 patients on vvECMO support (January 2006 to August 2017) who had been treated with UFH as primary anticoagulant. After propensity-score matching, the control group consisted of 78 patients receiving UFH (Fig. 1). The patient characteristics of the entire study cohort after propensity-score matching are presented in Table 1. Treatment with either Argatroban or UFH resulted in similar rates for renal replacement therapy (Argatroban 41% vs. UFH 40%, *p* = 0.405), immunosuppression (Argatroban 15% vs. UFH 18%, *p* = 0.728), and disseminated intravascular coagulation (Argatroban 3% vs. UFH 8%, *p* = 0.422). Further details and baseline respiratory parameters are provided in the supplement (Additional file 1: Table e7).

**Table 1:** Patient characteristics of the entire study cohort before and after propensity-score matching

Variables	Before matching			After matching			
	Argatroban n = 39	UFH n = 465	SMD	Argatroban n = 39	UFH n = 78	SMD	*p* value
Age, years	55 (46; 61)	53 (40; 62)	0.197	55 (46; 61)	56 (48; 63)	0.096	0.538
Female patients	10 (26)	138 (30)	0.194	10 (26)	27 (35)	0.151	0.325
BMI, kg/m^2^	27.7 (24.9; 34.0)	27.8 (24.5; 33.0)	0.006	27.7 (24.9; 34.0)	29.2 (24.9; 33.2)	0.095	0.675
SOFA	12.0 (10.0; 15.0)	12.0 (9.0; 15.0)	0.124	12.0 (10.0; 15.0)	12.0 (9.0; 14.3)	0.153	0.354
ARDS subgroup			0.549			0.284	0.816
I: pulmonary	31 (79)	281 (60)	–	31 (79)	60 (77)	–	
II: non-pulmonary	5 (13)	95 (20)	–	5 (13)	9 (11)	–	
III: post-trauma	0 (0)	43 (9)	–	0 (0)	2 (3)	–	
IV: mis-cellaneous	3 (8)	46 (10)	–	3 (8)	7 (9)	–	
Days of ventilation before ECMO	1 (0; 2)	1 (1;4)	0.212	1 (0; 2)	1 (0; 2)	0.146	0.774
Resuscitation before ECMO	8 (21)	74 (16)	0.119	8 (21)	15 (19)	0.032	0.869
Days on ECMO support	11 (8; 16)	8 (6; 14)	0.349	11 (8; 16)	10 (6; 16)	0.086	0.303

Data are expressed as n (%), median (25. percentile; 75. percentile); miscellaneous includes drowning, pulmonary embolism, cystic fibrosis, and bridge to transplant; ARDS: acute respiratory distress syndrome; UFH: unfractionated heparin; SMD: standardized mean difference

### Primary endpoint

In the primary analysis, major bleeding and/or major thrombosis occurred in 31 (79%) patients in the Argatroban group and in 65 (83%) in the UFH group (Additional file 1: Fig. e1). Non-inferiority was shown for Argatroban (upper limit of the 90%-CI < 10%) with an absolute risk difference of -4.9% (90%-CI − 15.6 to 7.9%; *p* = 0.026).

### Secondary endpoints

#### Thrombosis and bleeding

Major thrombosis (Argatroban 28% vs. UFH 17%, *p* = 0.145) and major bleeding events (Argatroban 69% vs. UFH 81% *p* = 0.163) were similarly distributed between the two groups (Table 2, Additional file 1: Fig. e1). All patients in the extrapulmonary ARDS group suffered from thrombosis and/or bleeding (Additional file 1: Table e8). Sensitivity analysis of major bleeding for a drop in hemoglobin of ≥ 2 g/dl/day (Argatroban 44% vs. UFH 51%, *p* = 0.433), transfusion of ≥ 2 packed red cells/24 h (Argatroban 44% vs. UFH 49%, *p* = 0.600), and bleeding according to body site (Argatroban 5% vs. UFH 15%, *p* = 0.107) showed similar results. More details are presented in the supplements (Additional file 1: Table e9).

**Table 2:** Comparison between the Argatroban and the unfractionated heparin group: secondary endpoints: thrombosis, bleeding, transfusion, and cerebral pathologies

Variables	Argatroban n = 39	UFH n = 78	*p* value
Major thrombosis	11 (28)	13 (17)	0.145
Pulmonary embolism on ECMO	2 (5)	3 (4)	0.747
ECMO days per oxygenator	9 (6; 12)	8 (6; 10)	0.210
Major bleeding	27 (69)	63 (81)	0.163
RBC transfusion per day on ECMO	0.3 (0; 0.5)	0.3 (0; 0.7)	0.287
FFP transfusion per day on ECMO	0.0 (0; 0)	0.0 (0; 0)	0.063
Platelet transfusion/day on ECMO	0.0 (0; 0)	0.0 (0; 0.1)	**0.044**
Cerebral hemorrhage	2 (5)	8 (10)^a^	0.584
Successful discharge from hospital	30 (77)	47 (60)	0.073
SOFA score at the end of ECMO support	6.0 (4; 8)	5.0 (3; 7)	0.058

Data are expressed as n (%), median (25. percentile; 75. percentile); UFH: unfractionated heparin group; major: thrombosis due to vein occlusion > 50%; major bleeding: drop in hemoglobin of ≥ 2 g/dl/day, and/or transfusion of ≥ 2 packed red cells/24 h, retroperitoneal, cerebral, or pulmonary bleeding; RBC: red blood cell; FFP: fresh frozen plasma

^a^One patient additionally suffered from cerebral ischemia; significant *p* values (*p* < 0.05) are marked in bold

Other analyses of the risks of coagulation such as pulmonary embolism and the lifespan of the oxygenator, minor thrombosis, and minor bleeding did not differ between the two groups (Table 2, Additional file 1: Table e10). Similar rates were also observed for cerebral hemorrhage (Table 2).

#### Transfusion and time-course of platelets

**Fig. 2:**
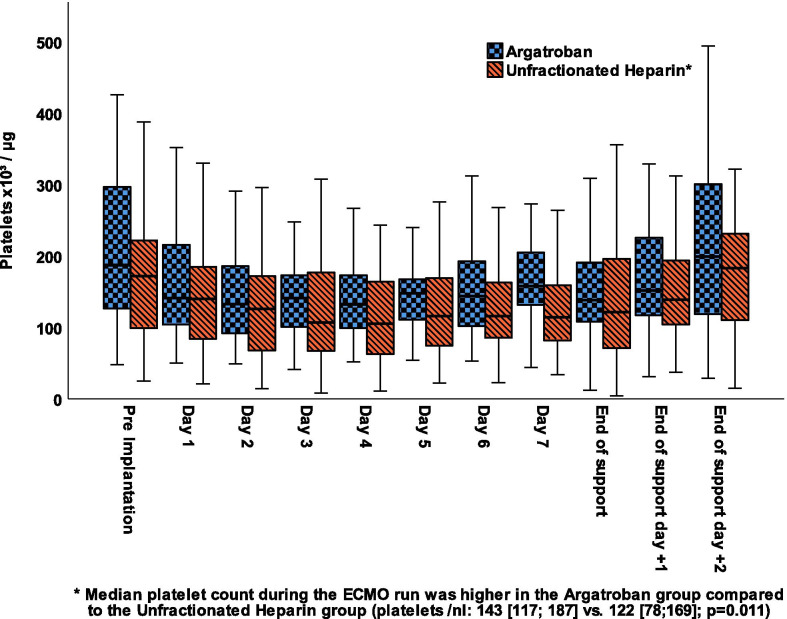
Box plot of the course of platelets throughout ECMO support according to anticoagulation. Data are expressed as median, minimum, maximum, 25. Percentile, and 75. Percentile

The amount of transfused RBC and FFP were equal between the two groups. The UFH group had received significantly more platelet transfusions (Table 2). The number of platelets was similar in both groups before ECMO therapy but lower in the UFH group after end of ECMO support (median [IQR]: 141 [104;198]/nl vs. 107 [54;171]/nl, *p* = 0.010; Fig. 2, Additional file 1: Table e11).

#### Technical complications and costs

Technical problems of the ECMO device were similarly distributed between the two groups (Table 3, Additional file 1: Table e12). Concerning isolated costs for anticoagulation therapy, Argatroban was more expensive per day on ECMO than UFH (median [IQR]: €26 [14; 53] vs. €0.9 [0.5; 1.5], *p* < 0.001). Expenses for substituted blood products per day during ECMO therapy were similar between Argatroban and UFH (median [IQR]: €28 [0; 65] vs. €34 [0; 152], *p* = 0.132). When additionally correcting for the diagnostic costs required in the heparin group to exclude heparin-induced thrombocytopenia, the expenses were aligning, but still tended to be higher in the Argatroban group (median [IQR] €63 [42; 171] vs. €40 [17; 158], *p* = 0.074; Table 4).

#### Monitoring of anticoagulation und laboratory values

**Table 3:** Technical complications in the Argatroban and the unfractionated heparin group modified according to ELSO categories (16)

Variables	Argatroban n = 39	UFH n = 78	*p* value
Technical complications in total	19 (49)	33 (42)	0.511
Thrombosis membrane oxygenator, thrombosis pump head and thrombosis cannula	11 (28)	19 (24)	0.653
Hyperfibrinolysis	13 (33)	21 (27)	0.472
Exchange of oxygenator necessary	16 (41)	31 (40)	0.894

Data are expressed as n (%), UFH: unfractionated heparin group

**Table 4:** Median costs in Euro of transfusions, anticoagulation, and heparin-induced-thrombocytopenia testing per day on ECMO according to the anticoagulation group

Variables	Argatroban n = 39	UFH n = 78	*p* value
Red blood cells	27.6 (0; 53.0)	26.7 (0; 72.6)	0.287
Fresh frozen plasma	0.0 (0.0; 0.0)	0.0 (0.0; 0.0)	0.063
Platelets	0.0 (0.0; 0.0)	0.0 (0.0; 44.4)	**0.044**
Blood products in total	28 (0.0; 65.4)	34 (0.0; 152.2)	0.132
Anticoagulation drugs	26 (13.8; 53.0)	0.9 (0.5; 1.5)	** < 0.001**
Blood products and anticoagulation drugs plus CLIA	63 (42; 171)^a^	40 (17; 158)	0.074
Blood products and anticoagulation drugs plus CLIA and HIPA	63 (42; 171)^a^	54 (25; 171)	0.364

Data are expressed as n (%), median (25. percentile; 75. percentile); transfusion costs are calculated as the number of transfusions in the group divided by the days on ECMO; costs are reported in Euro per day on ECMO

UFH: unfractionated heparin group; CLIA chemiluminescent immunoassay = rapid testing for heparin-induced thrombocytopenia; HIPA: heparin-induced platelet aggregation test.

^a^Testing for heparin-induced thrombocytopenia was not necessary; significant *p* values (*p* < 0.05) are marked in bold

Control of anticoagulation was similar with a median aPTT level of 52 [46; 57] sec in the Argatroban group vs. 49 [43; 57] sec in the UFH group, *p* = 0.189. Significantly more aPTT tests were conducted per day of ECMO therapy in the Argatroban group than in the UFH group (2.1 [1.7; 2.8] vs. 1.7 (1.4; 2.3], *p* = 0.004). Median Argatroban plasma concentration per day of ECMO was 0.29 µg/ml [0.14; 0.64]. In univariate analysis, Argatroban plasma concentration was moderately associated with the aPTT level (regression coefficient B [95% CI] 8.8 [6.5; 11.1], *p* < 0.001, Additional file 1: Fig. e2), increased during ECMO support, and was inversely correlated with lower SOFA scores at the end of ECMO therapy (B [95%-CI − 2.0 [− 3.5; − 0.4], *p* = 0.015). An anticoagulation threshold for thrombosis, bleeding, or a combination of both was neither observed for aPTT nor for Argatroban concentration (Additional file 1: Table e6). Median laboratory values between groups are presented in (Additional file 1: Table e13).

## Discussion

This study provides insights into the use of Argatroban as primary anticoagulant during vvECMO support with respect to safety, efficacy and complications in comparison to a propensity-score matched group of patients without HIT and anticoagulation with UFH.

In summary, Argatroban was non-inferior to UFH in view of clinically relevant complications such as major bleeding and major thrombosis. Similar results were seen for minor bleeding and minor thrombosis. Technical complications were also comparable. Of note, the decline in platelet count was more pronounced in the UFH group than in the Argatroban group despite receiving more platelet transfusions. Costs for the anticoagulant were substantially higher in the Argatroban group; yet, after accounting for blood transfusions and HIT testing in the UFH group, expenses were aligning.

### Complications

Anticoagulation during vvECMO therapy is much scrutinized because the device per se may cause both clotting and hemorrhagic complications [16]. Systemic anticoagulation is necessary to lessen potential lethal complications such as thromboembolism [4] but also increases the risk of bleeding [17, 18]. The ELSO anticoagulation guideline does not include any specific recommendations on anticoagulation during vvECMO support [19]. According to a recent survey, 264 out of 273 ECMO centers routinely use UFH as primary anticoagulant [20].

In the current analysis, Argatroban and UFH did not differ regarding the composite endpoint of major bleeding and major thrombosis. Although bleeding complications are common during ECMO support, the reported incidence varies between 0 and 91% [18, 21]. Besides potential differences in treatment algorithms, these variations are most likely due to differing definitions and methods for assessing bleeding [5, 18, 21]. In our study, the rate of bleeding complications was numerically relatively high but was mainly related to the chosen definition of bleeding according to the ELSO classification [5] that is liberal compared to other definitions [22, 23]. Considering only patients with either a drop in hemoglobin of ≥ 2 g/dl/day or a transfusion of ≥ 2 RBC/day, the incidence of major bleeding in our study was below 30%. The average number of RBC transfusions during ECMO support amounted to 0.3/day. Compared with data from a recent meta-analysis including 18 studies, this number is in the lower range [18]. In line with our results, Mazzeffi et al. reported that only the number of transfused blood products but not the bleeding episodes per se were associated with mortality during ECMO support [24]. Also, the incidence of cerebral hemorrhage in both our cohorts was low compared to other reports [25, 26]. The occurrence of bleeding, among others, depends on the pursued level of anticoagulation. Both our patient groups showed similar median aPTT levels of about 50 s throughout ECMO support. To accomplish this level, more aPTT checks per day were necessary in the Argatroban group. In our study, Argatroban doses had to be increased if markers for sepsis were dropping. We did not observe any clear threshold for aPTT or Argatroban-plasma concentration at which the rate of complications increased.

Severe bleeding with suggested standard dosing (2 µg/kg/min) of argatroban has been reported in critically ill patients on vvECMO [8]. Therefore, equally to others, initiating dose was set to 0.2 µg/kg/min in this study [8, 12]. In general, bleeding events are not uncommon during ECMO therapy [24]. Thus, the use of argatroban might be of advantage due to the shorter half-life time compared to UFH but in contrast to UFH an antidot is not available and the choice of how to monitor argatroban dosing is controversial [27]. Still, aPTT is commonly used for controlling anticoagulation with Argatroban [8, 11, 12, 28] and it also was shown that the target range of aPTT in vvECMO patients was more frequently achieved with argatroban compared to UFH [12].

Interestingly, the decline in the platelet count was more pronounced in the UFH group. This often described phenomenon of decreasing platelet numbers [5, 9] seems to be related to the ECMO device, possibly the pump unit, itself [29]. Whether heparin has an additional, independent effect on platelets—which may not occur with Argatroban—needs to be investigated in more detail, in particular due to the numerical inclusion imbalances between the Argatroban and the UFH group.

Besides bleeding, other complications with an important impact on outcome are thromboembolism [4, 30] and technical issues of the ECMO device caused by clotting. The incidence of thrombosis in patients requiring vvECMO support ranges from 0 to 85% [4]. Most previous studies reporting on the use of Argatroban during ECMO therapy included patients with type II HIT [8, 12]. However, HIT is associated with high rates of thromboembolism and hence lower survival rates [10]; therefore, a direct comparison with the current analysis that excludes patients with HIT is not feasible. The largest study on thromboembolism during vvECMO therapy to date reports incidences of any type of thrombosis of 62% [4], and the fraction of 28% clinically significant thromboembolic events was comparable to that of the Argatroban group in this study. Eventually, Argatroban may be an alternative in cohorts with a high risk for thromboembolism such as COVID-19 patients [31] due to its antithrombotic action, presumably also working in already formed clots, and its potential anti-inflammatory, and theoretical antiviral characteristics [32]. Technical complications related to clotting were similar. In 2004, Young et al. [33] found decreased thrombin generation in ECMO patients anticoagulated with Argatroban in comparison to UFH, but this observation has not been transferred into clinical practice so far.

It is likely that aPTT is not the best parameter to monitor anticoagulation with Argatroban and other parameters such as ecarin clotting time may be superior [28]. Yet, aPTT is widely available in clinical practice and has been frequently used to control anticoagulation with Argatroban in ECMO and non-ECMO patients [8, 11, 12, 34]. Alternatives such as Argatroban concentration or plasma-diluted thrombin time that are not influenced by various other factors such as D-Dimers and coagulopathy have no ceiling effect like aPTT and remain valid at higher ranges [10, 27, 35, 36]. In the current analysis, Argatroban concentrations only moderately correlated with aPTT levels. However, a therapeutic target range according to clinical efficacy has not yet been defined. In their case report, Kennedy et al. suggested a range of 0.4–1.2 μg/ml but did not provide any further evidence [37]. Technical complications such as pump or oxygenator failure were comparable between the two groups in our analysis and similar to previous reports [38]. By trend, successful discharge from hospital was higher in the Argatroban group; however, our study was not powered for outcome.

### Costs

Argatroban has been used for anticoagulation in patients with heparin-induced thrombocytopenia since two prospective studies in patients without ECMO showed a reduction in the composite endpoint (thrombosis, amputation, and death) in comparison to historical controls [11, 39]. Data from a retrospective study on patients with HIT showed a reduction in overall treatment costs despite the fact that absolute drug costs of Argatroban are 17 times higher than those of UFH, mainly because of reduced costs of transfusions [40]. Coughlin et al. [6] reported that overall costs of DTI are comparable to those of UFH after accounting for complications and additional tests. Likewise, the current study showed significantly higher direct drug costs in the Argatroban group, but costs for the two groups were aligning when including transfusions and HIT-testing. Thus, from an economical point of view, our results showed that Argatroban can be viewed as an alternative anticoagulant during vvECMO therapy, and not only for patients with HIT.

### Limitations

This single-center retrospective study was conducted by staff with considerable experience in the use of Argatroban. Therefore, some singularities of Argatroban were known before the initiation of the study, for instance the need of a very low initiation dose and a stepwise increase during the further treatment course. A direct causal relationship cannot be inferred due to the study’s design. Patients with cardiogenic shock during veno-arterial (va) ECMO therapy were excluded a priori to avoid cases of liver failure, for which Argatroban is contraindicated. A systematic bias may have occurred since the classification of bleeding events is limited in retrospective studies. To address this issue, we also reported the daily need of RBC transfusion. Our study included patients from a time period of 13 years. We cannot exclude that some details of management had changed over time; however, standards of care, persons in charge and technical set-up remained essentially the same so that outcome data are comparable to results previously published by our research group.

Finally, due to the limited number of patients treated with Argatroban, small effects and differences may have been missed, and future prospective studies should involve larger cohorts.

## Conclusion

Argatroban was non-inferior to UFH with respect to bleeding and thrombotic episodes in patients without HIT during vvECMO therapy. Technical complications were similarly distributed between the two treatment groups. Argatroban may have less impact on platelet decrease during ECMO; however, this result needs further evaluation. Direct drug costs were higher in the Argatroban group but comparable to the UFH group after accounting for HIT-testing and transfusions. In summary, the direct thrombin inhibitor Argatroban can be safely used as an alternative anticoagulant in patients during vvECMO therapy.

## Supplementary Information

**Additional file 1.** Supplementary information on the methods, anticoagulation strategy and further results.

## Data Availability

The datasets used and/or analyzed during the current study are available from the corresponding author on reasonable request.

## Abbreviations

aPTT: Activated partial thromboplastin time
ARDS: Acute respiratory distress syndrome
BMI: Body mass index
DIC: Disseminated intravascular coagulation
DTI: Direct thrombin inhibitors
ECMO: Extracorporeal membrane oxygenation
ELSO: Extracorporeal life support organization
FFP: Fresh frozen plasma
HIPA: Heparin-induced platelet aggregation
HIT: Heparin-induced thrombocytopenia
RBC: Red blood cells
SOFA: Sequential organ failure assessment
UFH: Unfractionated heparin
vvECMO: Veno-venous extracorporeal membrane oxygenation
