# Supplementary material for: Argatroban versus heparin in patients without heparin-induced thrombocytopenia during venovenous extracorporeal membrane oxygenation: a propensity-score matched study
Source: Crit Care. 2021 Apr 29;25:160. doi: 10.1186/s13054-021-03581-x (PMC8081564; doi:10.1186/s13054-021-03581-x)
Supplement: Supplementary file 1 — Additional file 1. Supplementary information on the methods, anticoagulation strategy and further results. [file 13054_2021_3581_MOESM1_ESM.docx]

**Supplemental material:**

This appendix has been provided by the authors to give readers additional information about the study.

Supplement to:

**Argatroban versus heparin in patients without heparin-induced thrombocytopenia during venovenous extracorporeal membrane oxygenation: a propensity-score matched study**

Christoph Fisser, MD, Maren Winkler, MD; Maximilian V. Malfertheiner, MD; Alois Philipp; Maik Foltan; Dirk Lunz, MD; Florian Zeman; Lars S. Maier, MD; Matthias Lubnow, MD^;^ Thomas Müller, MD

*Disseminated intravascular coagulation*

The disseminated intravascular coagulation (DIC) score [1] includes the level of prothrombin time, D-dimer, fibrinogen, and platelets and may vary from 0 to 7 points (Table e1). DIC was considered in the case of a total of ≥5 points. Instead of prothrombin time, Quick-values of <50% were rated with 2 points, values of <70% with 1 point and >70% with 0 points [2].

*ECMO implantation and anticoagulation*

At the time of cannulation, a bolus of up to 5000 units of unfractionated heparin was administered, depending on the respective circumstances and according to the provider’s recommendation (80 IE/kg). From September 2017 onwards, we used Argatroban (0.2µg/kg/min) with a target aPTT of 50±5 sec (Table e2, e3). Before September 2017, unfractionated heparin was applied according to provider’s recommendation (Table e4).

In the case of severe bleeding or a very low platelet count (<10/nl), anticoagulation was adapted until the event was resolved. In the case of severe bleeding, we administered fresh frozen plasma, tranexamic acid, platelets or prothrombin, or both. APTT levels were controlled every 2-4 hours [3] until target aPTT was achieved. In patients with stable aPTT levels, anticoagulation parameters were controlled at least once daily.

*Cost analysis*

Direct drug costs were assessed for unfractionated heparin and Argatroban. Drug costs during ECMO support were analyzed by means of the applied doses (in mg) of Argatroban in the Argatroban group and the international units of UFH in the UFH group.

Testing for heparin-induced thrombocytopenia was done with first antibodies against platelet factor 4 (HemosIL Acustar HIT-IgG, Werfen, Germany) and confirmed with HIPA (heparin-induced platelet aggregation; Department of Immunological and Transfusion Medicine University Hospital Greifswald). Costs are depicted in Table e5.

*Statistics*

The propensity-score matching algorithm was set to a 1:2 optimal matching with a caliper of 0.2 without replacement. The clinically acceptable non-inferiority margin of the primary composite endpoint (major bleeding and/or major thrombosis) was set to 10% according to the committee for medicinal products for human use guidelines [4] and clinical judgement of experts in the field of ECMO therapy with more than 15 years of experience in treating ECMO patients because comparable studies on this subject are lacking. Moreover, the maximal possible power was applied by using all patients eligible for this analysis and a post-hoc power analysis was not performed according to limitations previously described in literature [5]. Non-inferiority was calculated by means of the Farrington-Manning method. The quality of matching was assessed with the standardized mean difference (SMD). An SMD of <0.1 was considered optimal, and matching was considered successful when at least 50% of all SMDs were below 0.1.

**
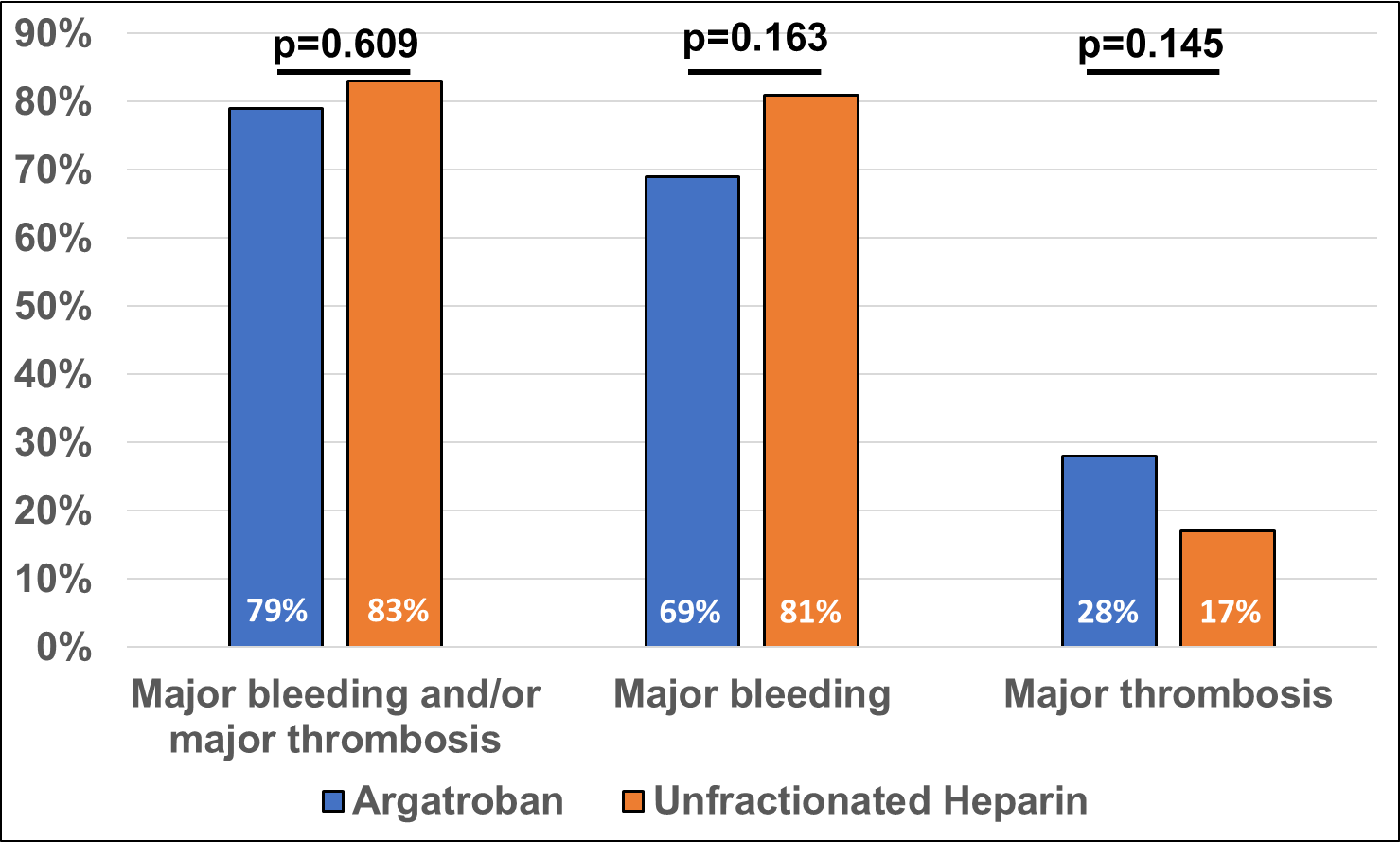
**Figure e1: Bar chart of primary and secondary endpoints (major bleeding and major thrombosis) according to anticoagulation. Data are expressed in percentage

Figure e2: Scatter plot of median Argatroban concentration vs median activated partial thrombin time


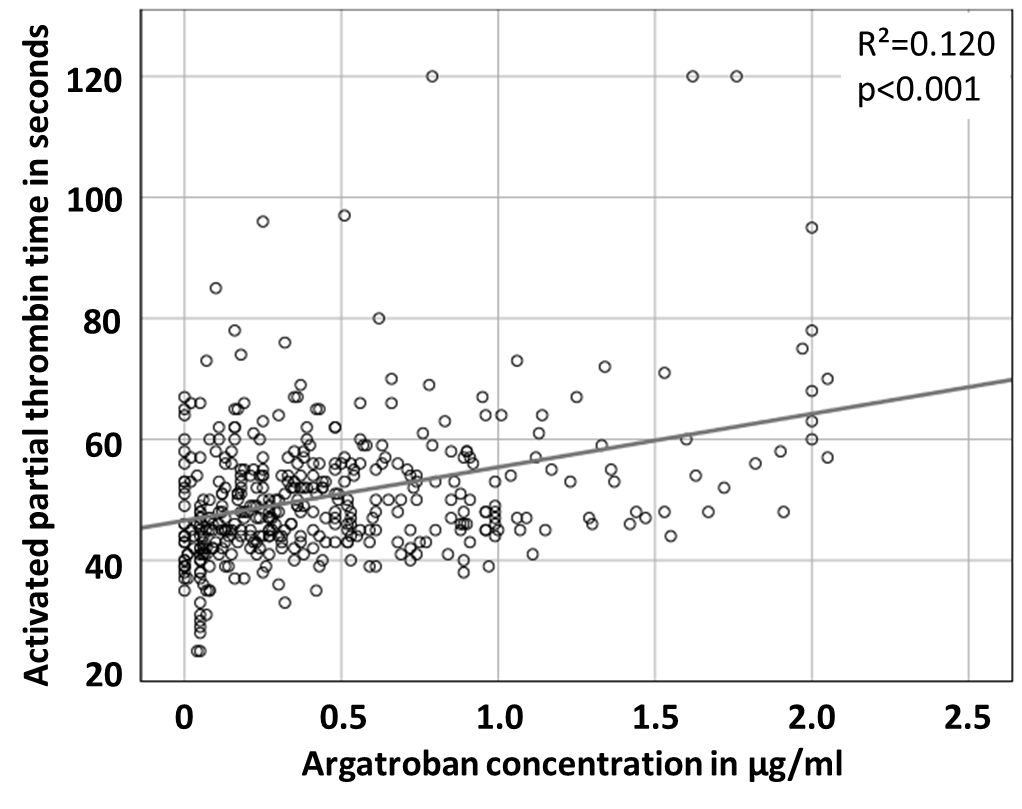


Table e1 Disseminated intravascular coagulopathy score

| Parameters | Values | Points |
| --- | --- | --- |
| Platelet count | >100 /nl | 0 |
|  | <100 /nl | 1 |
|  | <50 /nl | 2 |
| Fibrinogen | >1 g/l | 0 |
|  | <1 g/l | 1 |
| D-dimer | No increase (≤2 µg/ml) | 0 |
|  | Moderate increase (2.1-4.9 µg/ml) | 1 |
|  | Strong increase (≥5 µg/ml) | 2 |
| Quick values | ≥70% | 0 |
|  | 50.1-69.9% | 1 |
|  | ≤50% | 0 |

Table e2: Infusion rate of Argatroban according to body weight

|  | Infusion rate |  | Infusion rate |
| --- | --- | --- | --- |
| kg | **0.2 µg/kg/min** | kg | **0.2 µg/kg/min** |
| 45 | 0.5 | 125 | 1.5 |
| 50 | 0.6 | 130 | 1.6 |
| 55 | 0.7 | 135 | 1.6 |
| 60 | 0.7 | 140 | 1.7 |
| 65 | 0.8 | 145 | 1.7 |
| 70 | 0.8 | 150 | 1.8 |
| 75 | 0.9 | 155 | 1.9 |
| 80 | 1.0 | 160 | 1.9 |
| 85 | 1.0 | 165 | 2.0 |
| 90 | 1.1 | 170 | 2.0 |
| 95 | 1.1 | 175 | 2.1 |
| 100 | 1.2 | 180 | 2.2 |
| 105 | 1.3 | 185 | 2.2 |
| 110 | 1.3 | 190 | 2.3 |
| 115 | 1.4 | 195 | 2.3 |
| 120 | 1.4 | 200 | 2.4 |

Table e3: Argatroban infusion rate adapted according to provider’s information

| Initial dose | 0.2 µg/kg/min |
| --- | --- |
| aPTT, < 45 s | Increase infusion rate by 0.1 µg/kg/min |
| aPTT, 46-55 s | No change |
| aPTT, 56-70 s | Decrease infusion rate by 0.1 µg/kg/min |
| aPTT, 71-90 s | Decrease infusion rate by 0.2 µg/kg/min |
| aPTT, > 90 s | Hold infusion until target aPTT is reached, then decrease infusion rate by 50% |

aPTT: activated partial thromboplastin time

Table e4: Heparin infusion rate protocol adapted according to Raschke et al. [6]

| Initial dose | 80 units/kg bolus, then 18 units/kg/h |
| --- | --- |
| aPTT, < 35 s | 80 units/kg bolus, then increase 4 units/kg/h |
| aPTT, 35-45 s | 40 units/kg bolus, then increase 2 units/kg/h |
| aPTT, 46-55 s | No change |
| aPTT, 56-70 s | Decrease infusion rate by 1 units/kg/h |
| aPTT, 71-90 s | Decrease infusion rate by 2 units/kg/h |
| aPTT, > 90 s | Hold infusion 1 h, then decrease infusion rate by 3 units/kg/h |

aPTT: activated partial thromboplastin time

Table e5: Costs of anticoagulation and blood products in Euro

| Variable | Costs in Euro |
| --- | --- |
| Unfractionated heparin / international units | 0.000054 |
| Argatroban / mg | 0.756 |
| CLIA / test | 37.51 |
| HIPA / test | 89.76 |
| Packed red cells / unit | 105 |
| Fresh frozen plasma / unit | 75 |
| Platelets / unit | 400 |

Official acquisition costs are depicted in Euro [7]. CLIA chemiluminescent immunoassay = rapid testing for heparin-induced thrombocytopenia; HIPA: heparin-induced platelet aggregation test

Table e6: ROC-analysis: Area under the curve

| Variable | Thrombosis in total | Major thrombosis | Minor thrombosis | Bleeding in total | Major bleeding | Minor bleeding | Major thrombosis and bleeding |
| --- | --- | --- | --- | --- | --- | --- | --- |
| Median level of argatroban | 0.603 | 0.495 | 0.645 | 0.488 | 0.533 | 0.443 | 0.498 |
| Median aPTT | 0.438 | 0.399 | 0.532 | 0.581 | 0.608 | 0.407 | 0.513 |

ROC: receiver operating curve; aPTT: activated partial thrombin time; major: thrombosis due to vein occlusion >50%; minor: ≤50% of lumen diameter; major bleeding: drop in hemoglobin of ≥2g/dl/day or transfusion of ≥2 packed red cells/24 hours, retroperitoneal, cerebral or pulmonary bleeding; minor: <2g/dl/day or <2 packed red cells/24 hours

Table e7: Baseline respiratory characteristics between Argatroban and matched controls before ECMO initiation

|  | Argatroban  n = 39 | UFH  n = 78 | p-value |
| --- | --- | --- | --- |
| Minute ventilation in l/min | 10.0 (8.0; 12.0) | 10.0 (8.3; 12.5) | 0.611 |
| Tidal volume in ml | 469 (388; 560) | 476 (420; 567) | 0.583 |
| PEEP in mmHg | 15 (14; 16) | 15 (14;18) | 0.306 |
| Maximal inspiratory pressure in mbar | 33 (30; 36) | 33 (30; 38) | 0.785 |
| PaO2/FiO2 in mmHg | 82 (67; 115) | 63 (56; 84) | 0.001 |
| paCO2 in mmHg | 57 (50; 86) | 62 (53; 79) | 0.824 |
| pH | 7.20 (7.13; 7.30) | 7.22 (7.16; 7.32) | 0.321 |

Data are expressed as median (25. percentile; 75. percentile); UFH: unfractionated heparin group

Table e8: Primary endpoint according to ARDS subgroups stratified for group of anticoagulation

| Variable | Major bleeding and/or major thrombosis  **Yes** | Major bleeding and/or major thrombosis  **No** | p-value |
| --- | --- | --- | --- |
| UFH group | N=65 | N=13 | 0.120 |
| Pulmonary ARDS | 49 (75%) | 11 (85%) |  |
| Extrapulmonary ARDS | 9 (14%) | 0 (0%) |  |
| Post-trauma ARDS | 2 (3%) | 0 (0%) |  |
| Miscellaneous | 5 (8%) | 2 (15%) |  |
| Argatroban group | N=31 | N=8 | 0.433 |
| Pulmonary ARDS | 24 (77%) | 7 (88%) |  |
| Extrapulmonary ARDS | 5 (16%) | 0 (0%) |  |
| Post-trauma ARDS | 0 (0%) | 0 (0%) |  |
| Miscellaneous | 2 (7%) | 1 (12%) |  |

Table e9: Major bleeding events split according to the drop in hemoglobin of ≥2g/dl/day) or transfusion of ≥2 packed red cells/24 hours or body site between the Argatroban and the unfractionated heparin group

|  | Argatroban  n = 39 | UFH  n = 78 |
| --- | --- | --- |
| Drop in hemoglobin of ≥2g/dl/day | 9 (23) | 22 (28) |
| Transfusion of ≥2 packed red cells/24 hours | 10 (26) | 17 (22) |
| Site of bleeding | 0 (0) | 1 (1) |
| Drop in hemoglobin of ≥2g/dl/day and transfusion of ≥2 packed red cells/24 hours | 6 (15) | 12 (15) |
| Transfusion of ≥2 packed red cells/24 hours and site | 0 (0) | 5 (6) |
| Drop in hemoglobin of ≥2g/dl/day and site | 1 (3) | 2 (3) |
| Drop in hemoglobin of ≥2g/dl/day and transfusion of ≥2 packed red cells/24 hours, and site of bleeding | 1 (3) | 4 (5) |

Data are expressed as n (%). Major bleeding according to body site was defined according to ELSO criteria [8] as retroperitoneal, cerebral, or pulmonary bleeding.

Table e10: Complications between the Argatroban and the unfractionated heparin group:

Secondary endpoints: thromboembolism and bleeding

|  | Argatroban  n = 39 | UFH  n = 78 | p-value |
| --- | --- | --- | --- |
| Thrombosis | 22 (56) | 32 (41) | 0.116 |
| Minor thrombosis | 11 (28) | 19 (24) | 0.653 |
| Number of oxygenators | 1 (1.0; 2.0) | 1 (1.0; 2.0) | 0.926 |
| Hemorrhage in total | 34 (87) | 71 (91) | 0.518 |
| Minor bleeding | 7 (18) | 8 (10) | 0.241 |
| Total amount of transfusions per ECMO run | 3 (0; 6) | 4 (0; 13) | 0.151 |

Data are expressed as n (%), median (25. percentile; 75. percentile); UFH: unfractionated heparin group; minor thrombosis: ≤50% of lumen diameter

Table e11: Course of platelets throughout ECMO support differentiated between Argatroban and unfractionated heparin

| Variables | Argatroban  n = 39 | UFH  n = 78 | p-value |
| --- | --- | --- | --- |
| Pre implantation | 187 (124; 305) | 172 (98; 223) | 0.160 |
| Day 1 | 141 (103; 223) | 140 (80; 186) | 0.489 |
| Day 2 | 133 (92; 187) | 126 (64; 175) | 0.241 |
| Day 3 | 141 (99; 174) | 107 (66; 178) | 0.103 |
| Day 4 | 132 (97; 176) | 106 (63; 165) | 0.109 |
| Day 5 | 148 (109; 168) | 116 (73; 171) | 0.152 |
| Day 6 | 144 (100; 195) | 116 (85; 170) | 0.091 |
| Day 7 | 158 (126; 206) | 114 (80; 160) | 0.010* |
| End of support | 141 (104; 198) | 107 (54; 171) | 0.010* |
| End of support day 1 | 152 (117; 228) | 139 (101; 195) | 0.131 |
| End of support day 2 | 200 (118; 304) | 183 (110; 233) | 0.080 |

Data are expressed as median /nl, minimum, maximum, 25. percentile, and 75. percentile. Significant differences (p < 0.05) between Argatroban and unfractionated marked with *.

Table e12: Technical problems between the Argatroban and the unfractionated heparin group modified according to ELSO criteria [9]

| Variables | ARG  n = 39 | UFH  n = 78 | p-value |
| --- | --- | --- | --- |
| Oxygenator Failure | 9 (23) | 17 (22) | 0.875 |
| Pump Failure | 0 (0) | 5 (6) | 0.106 |
| Air in Circuit | 0 (0) | 2 (3) | 0.313 |
| System exchange | 1 (3) | 5 (6) | 0.374 |
| Cannula Problems | 0 (0) | 1 (1) | 0.478 |
| Defects of control console | 1 (3) | 2 (3) | 1.000 |

Data are expressed as n (%), UFH: unfractionated heparin group

Table e13: Laboratory values between the unfractionated heparin and the Argatroban group

| Variable | Argatroban  n = 39 | UFH  n = 78 | p-value |
| --- | --- | --- | --- |
| aPTT in seconds | 52 (46; 57) | 49 (43; 57) | 0.189 |
| International Normalized Ratio | 1.4 (1.3; 1.5) | 1.2 (1.1; 1.3) | <0.001 |
| Fibrinogen in mg/dl | 371 (274; 473) | 417 (305; 550) | 0.039 |
| Antithrombin III in % | 91 (77; 104) | 71 (57; 90) | <0.001 |
| Plasma free hemoglobin in mg/l | 36 (31;41) | 50 (36; 78) | <0.001 |
| Platelets /nl | 143 (117; 187) | 122 (78; 169) | 0.011 |
| GOT in U/l | 59 (37; 105) | 73 (38; 142) | 0.125 |
| GPT U/l | 44 (32; 66) | 47 (30; 98) | 0.149 |
| Bilirubin total in mg/dl | 0.7 (0.5; 1.5) | 1.2 (0.7; 2.2) | 0.041 |
| Creatinine in mg/dl | 1.25 (0.8; 1.6) | 1.3 (0.8; 1.8) | 0.416 |
| Urea in mg/dl | 69 (55; 101) | 67 (53; 97) | 0.440 |
| Alkaline Phosphatase in U/l | 89 (69; 168) | 103 (66; 154) | 0.986 |
| Hemoglobin in g/dl | 9.3 (8.8; 9.9) | 9.2 (8.7; 10.0) | 0.374 |
| D-Dimer in mg/l | 10 (7; 19) | 11 (7; 24) | 0.806 |
| Decline in D-Dimer after-ECMO ≤50%^a^ | 5 (13) | 10 (13) | 0.070 |

Data are expressed as n (%), median (25. percentile; 75. percentile); UFH: unfractionated heparin group; ^a^ comparison of decrease in D-dimer the day of and the day after decannulation

| STROBE Statement | Item No | Recommendation |
| --- | --- | --- |
| **Title and abstract** | 1 | (*a*) Indicate the study’s design with a commonly used term in the title or the abstract **[Within method section of the abstract page 2 ]** |
|  |  | (*b*) Provide in the abstract an informative and balanced summary of what was done and what was found **[See results section of abstract page 2, 3 ]** |
| Introduction | | |
| Background/rationale | 2 | Explain the scientific background and rationale for the investigation being reported **[page 4-5 ]** |
| Objectives | 3 | State specific objectives, including any prespecified hypotheses **[ page 4-5 ]** |
| Methods | | |
| Study design | 4 | Present key elements of study design early in the paper **[ page 6 ]** |
| Setting | 5 | Describe the setting, locations, and relevant dates, including periods of recruitment, exposure, follow-up, and data collection **[ page 6-8 ]** |
| Participants | 6 | (*a*) Give the eligibility criteria, and the sources and methods of selection of participants. Describe methods of follow-up **[ page 6 ]** |
|  |  | (*b*) For matched studies, give matching criteria and number of exposed and unexposed **[8, supplements]** |
| Variables | 7 | Clearly define all outcomes, exposures, predictors, potential confounders, and effect modifiers. Give diagnostic criteria, if applicable **[ page 7-8 ]** |
| Data sources/ measurement | 8 | For each variable of interest, give sources of data and details of methods of assessment (measurement). Describe comparability of assessment methods if there is more than one group **[ page 9-10 ]** |
| Bias | 9 | Describe any efforts to address potential sources of bias **[N/A]** |
| Study size | 10 | Explain how the study size was arrived at **[ page 7 ]** |
| Quantitative variables | 11 | Explain how quantitative variables were handled in the analyses. If applicable, describe which groupings were chosen and why **[ page 9-10 ]** |
| Statistical methods | 12 | (*a*) Describe all statistical methods, including those used to control for confounding **[ page 6-8 ]** |
|  |  | (*b*) Describe any methods used to examine subgroups and interactions **[ page 7-8 ]** |
|  |  | (*c*) Explain how missing data were addressed **[N/A]** |
|  |  | (*d*) If applicable, explain how loss to follow-up was addressed **[N/A]** |
|  |  | (*e*) Describe any sensitivity analyses **[ 10 ]** |
| Results | | |
| Participants | 13 | (a) Report numbers of individuals at each stage of study—eg numbers potentially eligible, examined for eligibility, confirmed eligible, included in the study, completing follow-up, and analysed **[ page 9 ]** |
|  |  | (b) Give reasons for non-participation at each stage **[ page 9 ]** |
|  |  | (c) Consider use of a flow diagram **[ Figure e1 ]** |
| Descriptive data | 14 | (a) Give characteristics of study participants (eg demographic, clinical, social) and information on exposures and potential confounders **[ page 9; table 1 ]** |
|  |  | (b) Indicate number of participants with missing data for each variable of interest **[N/A]** |
|  |  | (c) Summarise follow-up time (eg, average and total amount) **[N/A]** |
| Outcome data | 15 | Report numbers of outcome events or summary measures over time **[ page 9-11 ]** |
| Main results | 16 | (*a*) Give unadjusted estimates and, if applicable, confounder-adjusted estimates and their precision (eg, 95% confidence interval). Make clear which confounders were adjusted for and why they were included **[ page 9-11 ]** |
|  |  | (*b*) Report category boundaries when continuous variables were categorized **[ page 9-11 ]** |
|  |  | (*c*) If relevant, consider translating estimates of relative risk into absolute risk for a meaningful time period **[N/A]** |
|  |  | Other analyses **[N/A]**  Report other analyses done—eg analyses of subgroups and interactions, and sensitivity analyses **[ 10 ]** |
| Discussion | | |
| Key results | 18 | Summarise key results with reference to study objectives **[ page 12 ]** |
| Limitations | 19 | Discuss limitations of the study, taking into account sources of potential bias or imprecision. Discuss both direction and magnitude of any potential bias **[ page 16 ]** |
| Interpretation | 20 | Give a cautious overall interpretation of results considering objectives, limitations, multiplicity of analyses, results from similar studies, and other relevant evidence **[ page 12-16 ]** |
| Generalisability | 21 | Discuss the generalisability (external validity) of the study results **[ page 12-16 ]** |
| Other information | | |
| Funding | 22 | Give the source of funding and the role of the funders for the present study and, if applicable, for the original study on which the present article is based **[page 20-21 ]** |

References

1. Taylor FB, JR, Toh CH, Hoots WK, Wada H, Levi M. Towards definition, clinical and laboratory criteria, and a scoring system for disseminated intravascular coagulation. Thromb Haemost. 2001;86:1327–30.

2. Fisser C, Reichenbächer C, Müller T, Schneckenpointner R, Malfertheiner MV, Philipp A, et al. Incidence and Risk Factors for Cannula-Related Venous Thrombosis After Venovenous Extracorporeal Membrane Oxygenation in Adult Patients With Acute Respiratory Failure. Crit Care Med. 2019;47:e332-e339. doi:10.1097/CCM.0000000000003650.

3. GlaxoSmithKline. Argatroban package insert. 2005.

4. Brown D, Volkers P, Day S. An introductory note to CHMP guidelines: choice of the non-inferiority margin and data monitoring committees. Stat Med. 2006;25:1623–7. doi:10.1002/sim.2561.

5. Hoenig JM, Heisey DM. The abuse of power: the pervasive fallacy of power calculations for data analysis. The American Statistician. 2001;55:19–24.

6. Raschke RA, Gollihare B, Peirce JC. The effectiveness of implementing the weight-based heparin nomogram as a practice guideline. Arch Intern Med. 1996;156:1645–9.

7. Black A, Heimerl S, Oertli L, Wilczek W, Greinacher A, Spannagl M, et al. Implementation of a rapid HIT immunoassay at a university hospital - Retrospective analysis of HIT laboratory orders in patients with thrombocytopenia. Thromb Res 2017. doi:10.1016/j.thromres.2017.08.008.

8. Brogan TV, Lequier L, Lorusso R, MacLaren G, Peek GJ. Extracorporeal life support: The ELSO red book. 5th ed. Ann Arbor, Michigan: Extracorporeal Life Support Organization; 2017.

9. Extracorporeal life support organization. ECLS Registry Report: International Summary. 2019. https://www.elso.org/Registry/Statistics/InternationalSummary.aspx. Accessed 13 Jan 2020.
